# ENaC expression correlates with the acute furosemide‐induced K^+^ excretion

**DOI:** 10.14814/phy2.14668

**Published:** 2021-01-07

**Authors:** Niklas Ayasse, Peder Berg, Jens Leipziger, Mads Vaarby Sørensen

**Affiliations:** ^1^ Department of Biomedicine, Physiology Aarhus University Aarhus C Denmark; ^2^ Aarhus Institute of Advanced Studies Aarhus University Aarhus C Denmark

**Keywords:** ENaC, furosemide, K^+^ excretion, Na^+^ excretion

## Abstract

**Background:**

In the aldosterone‐sensitive distal nephron (ASDN), epithelial sodium channel (ENaC)‐mediated Na^+^ absorption drives K^+^ excretion. K^+^ excretion depends on the delivery of Na^+^ to the ASDN and molecularly activated ENaC. Furosemide is known as a K^+^ wasting diuretic as it greatly enhances Na^+^ delivery to the ASDN. Here, we studied the magnitude of acute furosemide‐induced kaliuresis under various states of basal molecular ENaC activity.

**Methods:**

C57/Bl6J mice were subjected to different dietary regimens that regulate molecular ENaC expression and activity levels. The animals were anesthetized and bladder‐catheterized. Diuresis was continuously measured before and after administration of furosemide (2 µg/g BW) or benzamil (0.2 µg/g BW). Flame photometry was used to measure urinary [Na^+^] and [K^+^]. The kidneys were harvested and, subsequently, ENaC expression and cleavage activation were determined by semiquantitative western blotting.

**Results:**

A low K^+^ and a high Na^+^ diet markedly suppressed ENaC protein expression, cleavage activation, and furosemide‐induced kaliuresis. In contrast, furosemide‐induced kaliuresis was greatly enhanced in animals fed a high K^+^ or low Na^+^ diet, conditions with increased ENaC expression. The furosemide‐induced diuresis was similar in all dietary groups.

**Conclusion:**

Acute furosemide‐induced kaliuresis differs greatly and depends on the a priori molecular expression level of ENaC. Remarkably, it can be even absent in animals fed a high Na^+^ diet, despite a marked increase of tubular flow and urinary Na^+^ excretion. This study provides auxiliary evidence that acute ENaC‐dependent K^+^ excretion requires both Na^+^ as substrate and molecular activation of ENaC.

## Graphical abstract

Integrated model of how molecular ENaC activity influences the magnitude of acute furosemide‐induced kaliuresis. High molecular ENaC activity, induced by high K^+^ or low Na^+^ intake, augments furosemide induced kaliuresis. Contrary, low molecular ENaC activity, induced by low K^+^ or high Na^+^ intake, is associated with a reduced acute furosemide‐induced kaliuresis.

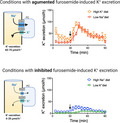

## 1 INTRODUCTION

Loop diuretics (eg, furosemide, bumetanide, and torsemide) antagonize the sodium, potassium, and two chloride cotransporter (NKCC2). NKCC2 is exclusively expressed in the apical membrane of the thick ascending limb (TAL) of the loop of Henle. In this water‐impermeable tubular segment, NKCC2 mediates the transcellular NaCl reabsorption that establishes the osmotic gradient allowing for the production of hyperosmotic urine (Mutig, 2017). Besides inducing severe diuresis and urinary NaCl loss, loop diuretics are also known to cause urinary K^+^ wasting. The diuretic and natriuretic effects of loop diuretics are used in clinical practice to reduce blood pressure and manage edema secondary to heart failure, hepatic, or renal diseases (Wargo & Banta, 2009). Urinary K^+^ loss is a severe adverse effect of long‐term administration of loop diuretics causing hypokalemia (Oh & Han, 2015). Clinical guidelines strive to correct loop diuretic‐induced hypokalemia by K^+^ supplementation (Clase et al., 2020; Cohen et al., 1987). Nevertheless, titration of K^+^ supplement to match loop diuretic‐induced K^+^ loss in the individual patient is a challenging task. This is highlighted in a study following almost 20,000 Danish heart failure patients on long‐term loop diuretic treatment. Approximately 20% of these patients showed life‐threatening hyperkalemia and another 5% displayed life‐threatening hypokalemia despite the clinical effort to manage plasma K^+^ levels by dietary K^+^ supplement (Aldahl et al., 2017).

Besides the use of loop diuretics to reduce the circulatory volume, they are also a clinical strategy to manage acute hyperkalemia (Long et al., 2018; Sterns et al., 2016). However, predicting the effective treatment dose versus the risk of water and electrolyte depletion of the individual patient is very difficult (Long et al., 2018).

Both examples highlight the importance of a better understanding of the principal mechanisms of loop diuretic‐induced K^+^ loss. An improved understanding of the physiological variability can help reduce the risk of life‐threatening K^+^ homeostatic disturbances when treating hyperkalemia with loop diuretics.

Electrogenic Na^+^ absorption in the late distal convoluted tubule (DCT), the connecting tubule (CNT), and the collecting duct (CD) is mediated by the epithelial sodium channel (ENaC), accounting for the reabsorption of around 1%–3% of the filtered Na^+^ (Staruschenko, 2012). Besides the direct contribution to overall Na^+^ homeostasis, ENaC activity is crucial for distal tubular K^+^ excretion via apical K^+^ channels. Electrogenic ENaC‐mediated Na^+^ absorption causes apical membrane depolarization that generates a necessary driving force for cellular K^+^ exit (Welling, 2016). Rare genetic diseases highlight the importance of sufficient ENaC function in the control of K^+^ homeostasis. Liddle syndrome, characterized by a gain of function mutation of ENaC, clinically presents with congenital severe hypertension and K^+^ wasting (Tetti et al., 2018). In contrast, pseudohypoaldosteronism type 1, characterized by loss of function mutations of ENaC, manifests with severe hypotension and K^+^ retention (Tajima et al., 2017). According to the current understanding, aldosterone‐sensitive distal nephron (ASDN)‐mediated K^+^ excretion is regulated by the delivery of Na^+^ to the CNT and CD. Plasma K^+^ itself controls the amount of Na^+^ delivered to the site of ENaC by direct regulation of the Na^+^/Cl^‐^ symporter (NCC), located to the DCT. That is, NCC is turned on and off when K^+^ needs to be conserved or excreted, respectively (Penton et al., 2016; Sorensen et al., 2013; Terker et al., 2016).

Thus, the delivery of Na^+^ to the site of ENaC is presumed to be one key factor controlling the magnitude of K^+^ excretion in the ASDN. Loop diuretics inhibiting NKCC2 in the TAL, which account for the absorption of around 20%–25% of the filtered Na^+^ (Castrop & Schiessl, 2014), greatly increase the amount of Na^+^ delivered to the site of ENaC. An instrumental study performed in rats by Hropot et al. (1985) documented the concurrent application of loop diuretics and ENaC channel blockers abolished the kaliuretic effect of loop diuretics. This suggests that ENaC activity is a prerequisite of loop diuretic‐induced kaliuresis.

These considerations presume that a sufficient a priori activity of ENaC will increase Na^+^ absorptive currents when more Na^+^ as substrate is available. A priori, molecular expression of ENaC is regulated by plasma aldosterone. Under conditions of low endogenous aldosterone, ENaC‐mediated Na^+^ reabsorptive capacity is reduced (Bhalla & Hallows, 2008).

In the present study, we hypothesized that the acute kaliuretic effect of furosemide varies significantly under different baseline ENaC activity levels, thus indicating that an increased Na^+^ delivery to the ASDN alone is not sufficient to induce acute K^+^ secretion. To further highlight the difference between functional and molecular ENaC activity, we investigated the effect of the ENaC inhibitor benzamil on natriuresis under control and low Na^+^ diet conditions.

## 2 METHODS

### 2.1 Animals and feeding protocols

**FIGURE 1:**
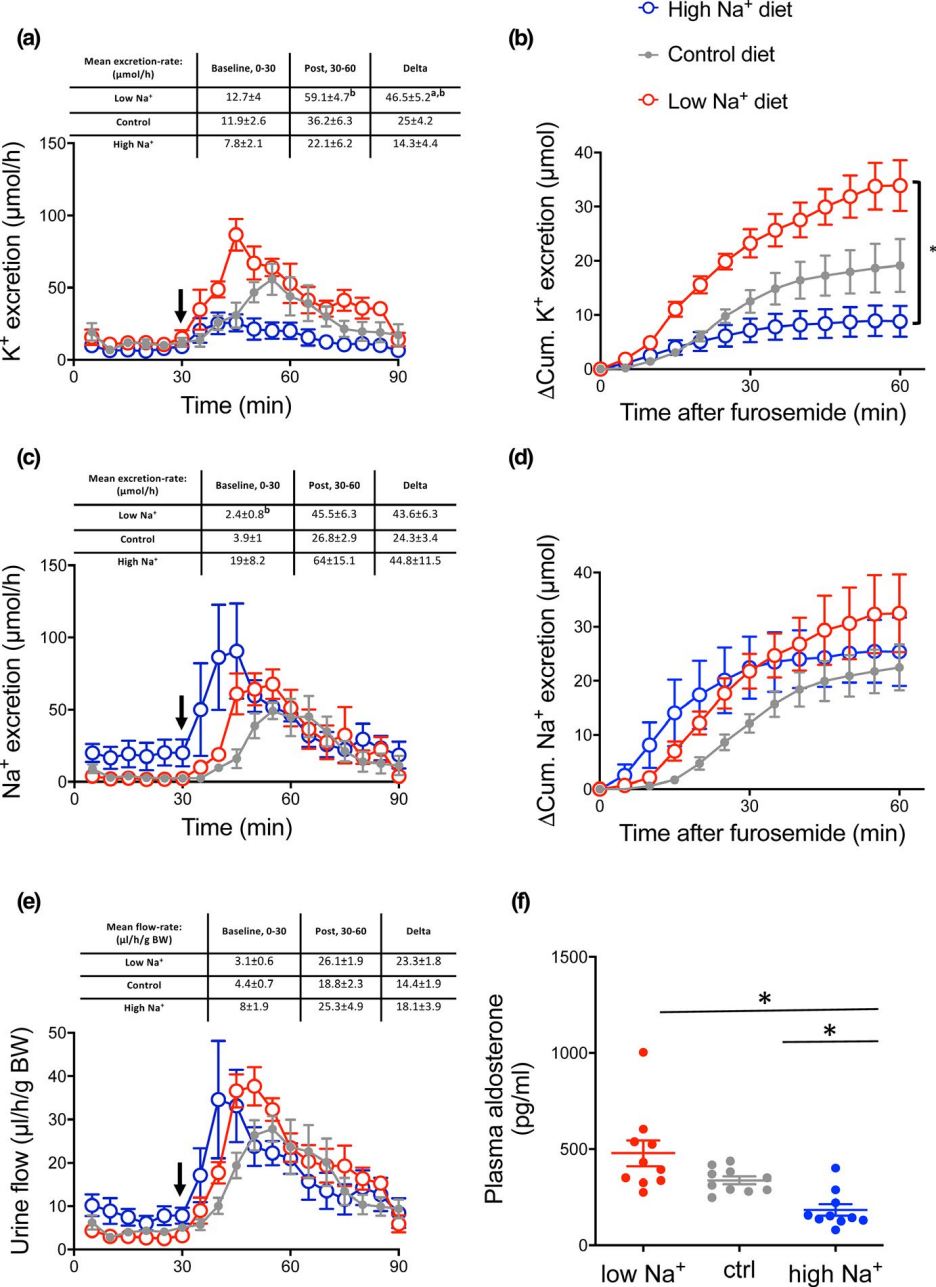
Furosemide induced kaliuresis, natriuresis and diureses in mice kept on high, control and low Na^+^ diet. Black arrows indicate time of furosemide injection. (a) K^+^ excretion rate. Graph display real time K^+^ excretion rate in 5 min intervals in control and dietary Na^+^ loaded and Na^+^ restricted mice. The values in the table represent K^+^ excretion rates in 30 min intervals for statistical analyzes (15‐30 baseline and 30–60 the first half hour post furosemide injection). Note that the furosemide‐induced increase in kaliuresis was augmented in Na^+^ restricted mice and reduced in Na^+^ loaded mice. (b) 15–30 min baseline subtracted cumulative (ΔCum) K^+^ excretion after furosemide injection. Cumulated K^+^ excretion post furosemide treatment was increased in Na^+^ restricted mice. (c) Na^+^ excretion rate. Graph display real time Na^+^ excretion rate in 5 min intervals in control and dietary Na^+^ loaded and Na^+^ restricted mice. The values in the table represent Na^+^ excretion rates in 30 min intervals for statistical analyzes (15‐30 baseline and 30–60 the first half hour post furosemide injection). Baseline natriuresis was augmented in Na^+^ loaded mice compared to Na^+^ restricted mice. (d) 15–30 min baseline subtracted cumulative (ΔCum) natriuresis post furosemide injection. Cumulated natriuresis was increased in Na^+^ loaded mice. (e) Urine output. Graph display real time diuresis in 5 min intervals in control and dietary Na^+^ loaded and Na^+^ restricted mice. The values in the table represent diuresis in 30 min intervals for statistical analyzes (15–30 baseline and 30–60 the first half hour post furosemide injection) measured 30 prior and 60 min post furosemide. A robust furosemide‐induced diuresis was measured in all groups. Differences between dietary groups were evaluated by comparing the men 15‐30 min. Baseline values, the difference between mean baseline and post‐treatment values and the cumulative excretion after furosemide treatment and analyzed by one‐way ANOVA followed by Bonferroni's multiple comparisons test. Tables above panels show mean values ± *SEM*. ‘a’ indicates a significant difference compared to the control group, ‘b’ indicates a significant difference compared to the high Na^+^ group. *n* = 5–6

All experiments were conducted in C57/Bl6J mice purchased from Janvier Labs. The mice were transported to Aarhus by a temperature‐controlled (21°C) car and acclimatized to the housing facility in Aarhus for at least 1 week before experimental use. All mice were kept in a room with a 12‐hr light/dark cycle and had ad libitum access to chow and water. The animals were randomly allocated to different diet groups. To augment plasma aldosterone concentration (Figures 1f and 3f) and ENaC expression, the mice were fed either a low Na^+^ diet (<0.03% Na^+^) for 1 week or a high K^+^ diet (5%) for 4 days. The low Na^+^ diet contained <0.03% Na^+^ and 0.97% K^+^ and was premade (ssniff Spezialdiäten GmbH). The high K^+^ diet was made by the addition of KCl to a ssniff EF R/M control diet (0.2% Na^+^) (ssniff, Spezialdiäten GmbH). To reduce plasma aldosterone concentration (Figures 1f and 3f) and ENaC expression, the animals were fed either a high Na^+^ diet (2%) for 4 days or a low K^+^ diet for 3 days. The high Na^+^ diet was made by adding NaCl to a ssniff EF R/M control diet (ssniff, Spezialdiäten GmbH). The low K^+^ diet was premade (<0.03% K^+^ and 0.22% Na^+^ Altromin C1037). The control group was fed ssniff EF R/M control diet (0.9% K^+^ and 0.2% Na^+^) (ssniff Spezialdiäten GmbH). The handling of the animals complied with the Danish animal welfare regulations (Animal experiment license from the Danish Animal Welfare Regulation Authority: 2017‐15‐0201‐001166).

### 2.2 Real‐time measurement of diuresis in vivo

An intraperitoneal (IP) bolus injection of a ketamine (10 mg/ml)/xylazine (1 mg/ml) mix at a dose of 0.1 ml/10 g BW was administered to induce anesthesia in the animals. Anesthesia was maintained by continuous intravenous anesthetic infusion at a rate of 0.03 ml/10 g/BW/hour of the abovementioned ketamine/xylazine solution.

A small incision in the lower abdomen was made and a custom‐made catheter was inserted in the urinary bladder. Urine was collected every 5 min from the outflow of the catheter, allowing real‐time measurement of diuresis and electrolyte excretion. The collected urine was immediately stored at −20°C. After a baseline period of 30 min, an IP of furosemide (2 µg/g BW) was administered and diuresis measurement was continued for another 60 min. In one experimental series, animals fed either a control or a low Na^+^ diet were given an IP benzamil bolus (0.2 µg/g BW) after 90 min and diuresis was measured for another 60 min.

### 2.3 Flame photometry

Urinary [Na^+^] and [K^+^] were measured with a flame photometer (420 Flame Photometer, Sherwood, UK) as described previously (Jensen et al., 2016), and Na^+^ and K^+^ excretion rates were calculated from the diuresis data.

### 2.4 Western blot

At the end of the functional experiments, the mice were sacrificed and kidneys were harvested for western blots as described in Larsen et al. (2016). Shortly, western blot was done on the supernatant of half kidneys, processed in lysis buffer in a TissueLyser in 30 s (Qiagen) and spun for 15 min (1000G).

Protein concentrations were measured using Pierce^TM^ BCA Protein Assay Kit and controlled by Coomassie. All samples were run on criterion TGX^TM^ Precast Gels (Bio‐rad). Ten microgram of total protein was administered to each sample dwell. Figure S1 shows the linear correlation between the amounts of protein loaded and detected signal for the used NKCC2 antibodies. Linear detection range validation of other used antibodies has been reported previously (Jensen et al., 2016). Membranes were developed using Clarity^TM^ Western ECL substrate (Bio‐rad) in an ImageQuant LAS 4000 mini (GE Healthcare Life Science). Density signals of the proteins of interest were normalized to the signal of a Coomassie staining of the membranes post chemiluminescent detection of the protein of interest (Welinder & Ekblad, 2011), to enable corrections for differences in protein loading. All images were analyzed using Image Studio^TM^ Lite (Li‐Cor). Antibody dilutions and sources are shown in Table 1.

**TABLE 1:** Information of origin and used dilutions of the antibodies.

Antibody	Host	µg protein loaded	Dilution	Source	Reference
α‐ENaC	Rabbit	10	1,000	Loffing, Zürich, Switzerland	Sorensen et al., 2013)
γ‐ENaC	Rabbit	10	7,500	Loffing, Zürich, Switzerland	Wagner et al., 2008)
tNCC	Rabbit	10	10,000	Loffing, Zürich, Switzerland	Sorensen et al., 2013)
pT53 NCC	Rabbit	10	5,000	Loffing, Zürich, Switzerland	Sorensen et al., 2013)
tNKCC2	Rabbit	10	500	Mutig, Berlin, Germany	Mutig et al., 2007)
pNKCC2	Rabbit	10	20,000	Mutig, Berlin, Germany	Mutig et al., 2007)

### 2.5 Analysis and statistics

All statistical analyses were performed in GraphPad Prism 7 (GraphPad Software). The distribution of data was evaluated by the generation of QQ‐plots. Datasets that did not follow a Gaussian distribution were log‐transformed before statistical analysis. In all cases, this resulted in a good approximation of a Gaussian distribution. For paired differences, also Bland‐Altman plots were generated to evaluate whether the variance and the mean of differences were dependent on the average value. For tests with an assumption of equal *SD* among groups, this was assessed by Brown‐Forsythe and Bartlett's test. Within‐group comparisons were tested with paired *t*‐tests. Comparisons between more than two groups were performed with one‐way ANOVA with Bonferroni's multiple comparison test. If both sample size and *SD* differed significantly between groups, comparisons with more than two groups were performed with Brown‐Forsythe and Welch ANOVA with Bonferroni's multiple comparison test. All tests were two‐sided and performed at a significance level of 5%. The statistical test used is stated in each figure legend. The same control diet group is plotted in Figures 1 and 3, but the five different diet groups were compared together with adjustment for multiple comparisons. Time‐dependent data are shown as mean ± *SEM*. Time‐independent data are shown as individual data points in dot plots with mean ± *SEM*. Graphical abstract was created with BioRender.com.

## 3 RESULTS

### 3.1 Furosemide‐induced kaliuresis is increased during Na^+^ restriction and markedly decreased by Na^+^ loading

The kaliuretic, natriuretic, and diuretic effects of furosemide were investigated in mice fed diets with differential Na^+^ content. Baseline (first 30 min) K^+^ excretion was similar in the high Na^+^‐, control‐, and low Na^+^ diet groups. Following furosemide administration, a strong kaliuretic response was detected in the control and Na^+^ restricted mice but almost absent in the Na^+^ loaded mice (Figure 1a). The furosemide‐induced increase in K^+^ excretion, measured as the difference in mean kaliuresis 15 min pre‐ and 30 min post‐furosemide injection, was almost 2‐fold larger in the Na^+^ restricted mice as compared to control mice (46.5 ± 5.2 vs. 25.0 ± 4.2 µmol/h, respectively) and 3‐fold increased as compared to Na^+^ loaded mice (46.5 ± 5.2 vs. 14.3 ± 4.4 µmol/h, respectively) (Figure 1a). The cumulated baseline subtracted K^+^ excretion in the first hour following furosemide treatment was significantly augmented in Na^+^ restricted mice as compared to Na^+^ loaded mice (Figure 1b).

Furosemide increased natriuresis in all experimental groups (Figure 1c). The cumulated baseline subtracted natriuresis following furosemide injection was equal between the experimental groups (Figure 1d). Furosemide is known to be a potent diuretic. A strong diuretic response to furosemide was observed in all diet groups (Figure 1e).

### 3.2 Molecular evidence for functional regulation of renal Na^+^ transporters by dietary Na^+^ content

**FIGURE 2:**
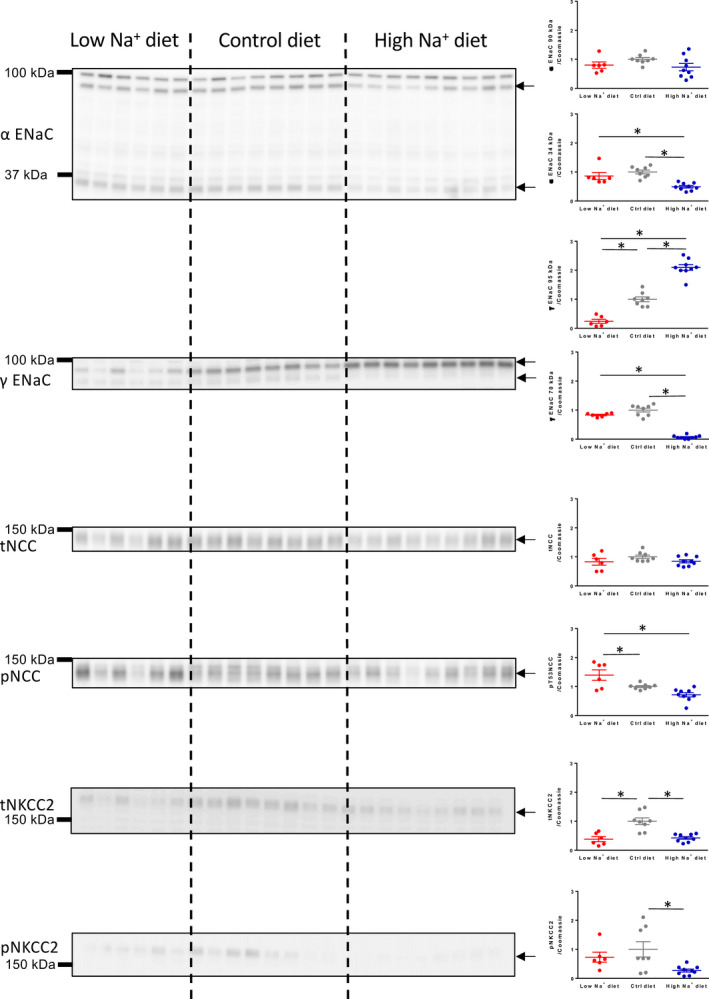
Semi‐quantitative immunoblots of full length (~90 kDa) and cleavage activated (~34 kDa) α‐ENaC, full length (~95 kDa) and cleavage activated (~70 kDa) γ‐ENaC, total and phosphorylated NCC and total and phosphorylated NKCC2 in kidneys from mice kept on low, normal and high Na^+^ diet. Reduced cleavage activation of both α‐ENaC and γ‐ENaC was detected in the high Na^+^ diet group whereas low Na^+^ diet augmented the amount of phosphorylation‐activated NCC. Arrows mark quantified bands. Panels with equal variance between groups were analyzed by one‐way ANOVA. Panels with unequal variance between groups were analyzed by Brown‐Forstythe and Welch ANOVA. Both were followed by Bonferroni's multiple comparisons test. *n* = 6–9. *indicates *p* < .05

Semiquantitative western blotting of distal tubular Na^+^ absorptive proteins showed a clear regulation pattern as a function of dietary Na^+^ loading (Figure 2). Full‐length (~90 kDa) α‐ENaC was unchanged by the dietary regimes. Importantly, proteolytically cleaved (~34 kDa) and thus activated α‐ENaC (Ergonul et al., 2006; Kleyman et al., 2006) was significantly less expressed in kidneys from Na^+^ loaded mice as compared to kidneys from control and Na^+^ restricted mice. The amount of full‐length (~95 kDa) γ‐ENaC correlated with the dietary Na^+^ content. It was significantly reduced in kidneys from Na^+^ restricted mice and increased in kidneys from Na^+^ loaded mice. Furthermore, the amount of the proteolytic‐activated (~70 kDa) γ‐ENaC (Ergonul et al., 2006; Frindt et al., 2008; Kleyman et al., 2006) was reduced in kidneys from the Na^+^ loaded mice when compared to kidneys from control and Na^+^ restricted mice.

The different Na^+^ diets did not change the expression of total NCC, but during Na^+^ restriction, we measured a significant increase in phosphorylation‐activated NCC at phosphor‐threonine residue 53 (Dimke, 2011) in the Na^+^ restricted mice.

The furosemide target NKCC2 was significantly reduced at the total protein level in kidneys from both the low Na^+^ diet and the high Na^+^ diet groups. However, the phosphorylation‐activated NKCC2 at phosphor‐threonine residues 96 and 101 (Gimenez & Forbush, 2005) was only reduced in kidneys from the Na^+^ loaded mice.

Taken together, the presented data suggest that the acute kaliuretic effect of a single furosemide dose is reduced under the conditions of low baseline ENaC activity and that furosemide‐induced natriuresis is augmented during low‐molecular NCC and ENaC activity despite reduced NKCC2 phosphorylation.

### 3.3 Furosemide‐induced kaliuresis is increased in mice fed a high K^+^ diet

The effect of furosemide was investigated under different levels of dietary K^+^ loading. To permit comparison, the control curves shown in Figure 1 are also presented.

**FIGURE 3:**
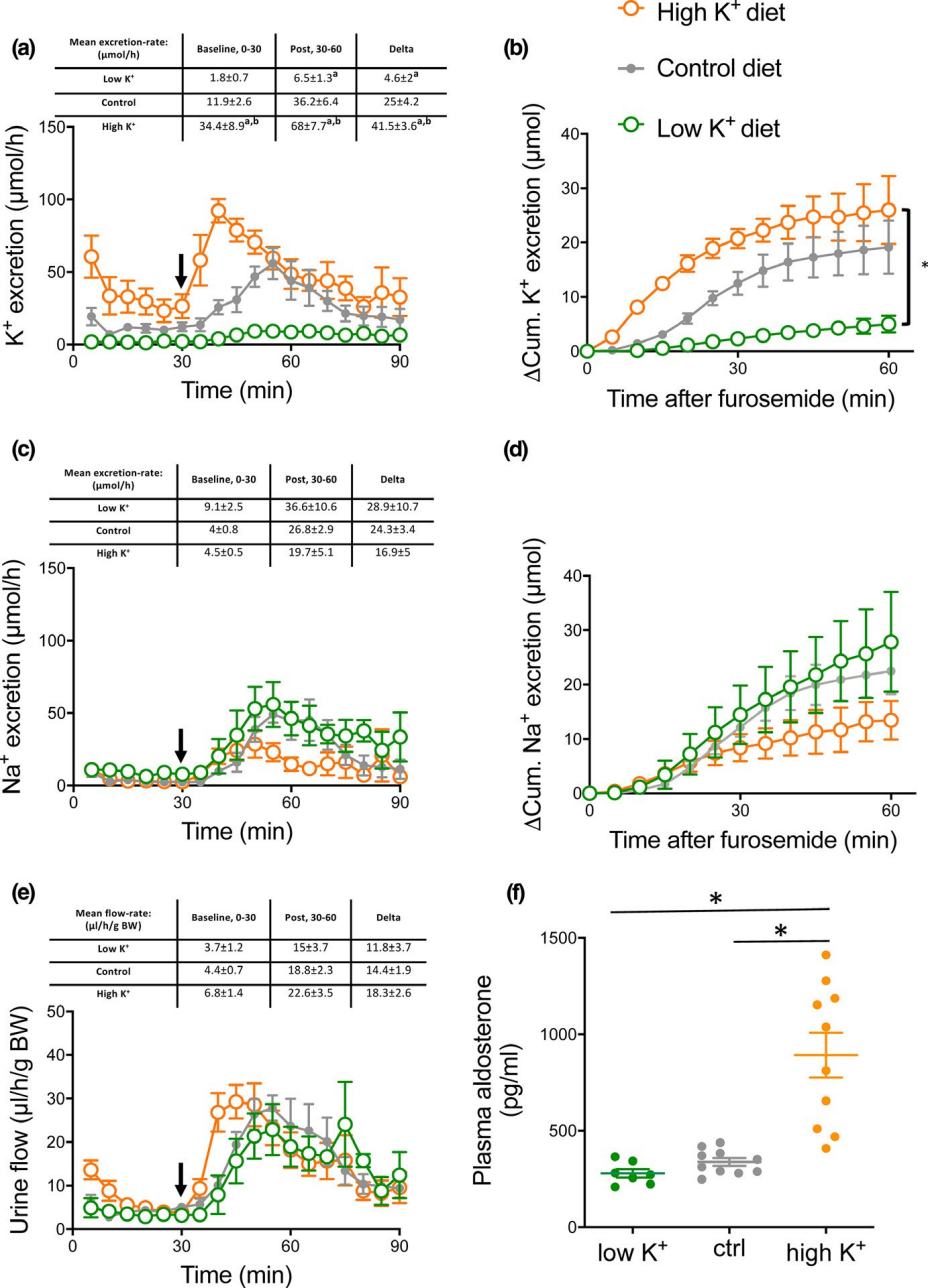
Furosemide‐induced kaliuresis, natriuresis and diureses in mice kept on high, control and low dietary K^+^ content. Black arrows indicate time of furosemide injection. (a) K^+^ excretion rate. Graph display real time K^+^ excretion rate in 5 min intervals in control and dietary K^+^ loaded and K^+^ restricted mice. The values in the table represent K^+^ excretion rates in 30 min intervals for statistical analyzes (15–30 baseline and 30–60 the first half hour post furosemide injection). Baseline kaliuresis was higher in K^+^‐loaded mice compared to control mice and K^+^ restricted mice. Note that the furosemide‐induced increase in kaliuresis was augmented in K^+^ loaded mice and reduced in K^+^ restricted mice. (b) 15–30 min baseline subtracted cumulative (ΔCum) K^+^ excretion after furosemide injection. Cumulated K^+^ excretion post furosemide treatment was increased in K^+^ loaded mice and decreased in K^+^ restricted mice. (c) Na^+^ excretion rate. Graph display real time Na^+^ excretion rate in 5 min intervals in control and dietary K^+^ loaded and K^+^ restricted mice. The values in the table represent Na^+^ excretion rates in 30 min intervals for statistical analyzes (15–30 baseline and 30–60 the first half hour post furosemide injection) The furosemide‐induced increase in natriuresis was almost 2‐fold lower in the K^+^ loaded mice, though not statistical different. (d) 15–30 min baseline subtracted cumulative (ΔCum) natriuresis post furosemide injection (e) Urine output. Graph display real time diuresis in 5 min intervals in control and dietary K^+^ loaded and K^+^ restricted mice. The values in the table represent diuresis in 30 min intervals for statistical analyzes (15–30 baseline and 30–60 the first half hour post furosemide injection) measured 30 prior and 60 min post furosemide. A robust furosemide‐induced diuresis was measured in all groups, which seemed slightly enlarged in the mice kept on a high K^+^ diet. Differences between dietary groups were evaluated by comparing the mean 30 min. baseline values, the difference between mean baseline and post‐treatment values and the cumulative excretion after furosemide treatment and analyzed by one‐way ANOVA followed by Bonferroni's multiple comparisons test. Tables above panels show mean values ± *SEM*. ‘a’ indicates a significant difference compared to the control group, ‘b’ indicates a significant difference compared to the low K^+^ group. *n* = 5–6

Baseline K^+^ excretion was significantly greater in the high K^+^ diet group as compared to the control‐ and the low K^+^ diet groups (Figure 3a).

Following furosemide administration, a kaliuretic response was detected in control and K^+^ loaded mice, which was almost absent in the K^+^ restricted mice. The furosemide‐induced increase in K^+^ excretion was approximately 2‐fold larger in the K^+^ loaded mice and virtually absent in the K^+^ restricted mice as compared to control mice (41.5 ± 3.6 vs. 25.0 ± 4.2 vs. 4.6 ± 2.0 µmol/h, respectively) (Figure 3a). Also, the baseline subtracted cumulated K^+^ excretion after furosemide treatment was significantly higher in the K^+^ loaded mice and significantly reduced in the K^+^ restricted mice as compared to control mice (Figure 3b).

Furosemide induced a significant increase in natriuresis in all experimental groups. The increase in natriuresis following furosemide injection was seemly lower in the K^+^ loaded mice compared to control and K^+^ restricted mice (Figure 3c). Figure 3d depicts the baseline subtracted cumulative Na^+^ excretion the first hour after furosemide injection. No significant differences were measured. Likewise, the furosemide‐induced diuresis was similar across the different dietary groups (Figure 3e).

### 3.4 Molecular evidence for functional regulation of renal Na^+^ transporters by dietary K^+^ content

**FIGURE 4:**
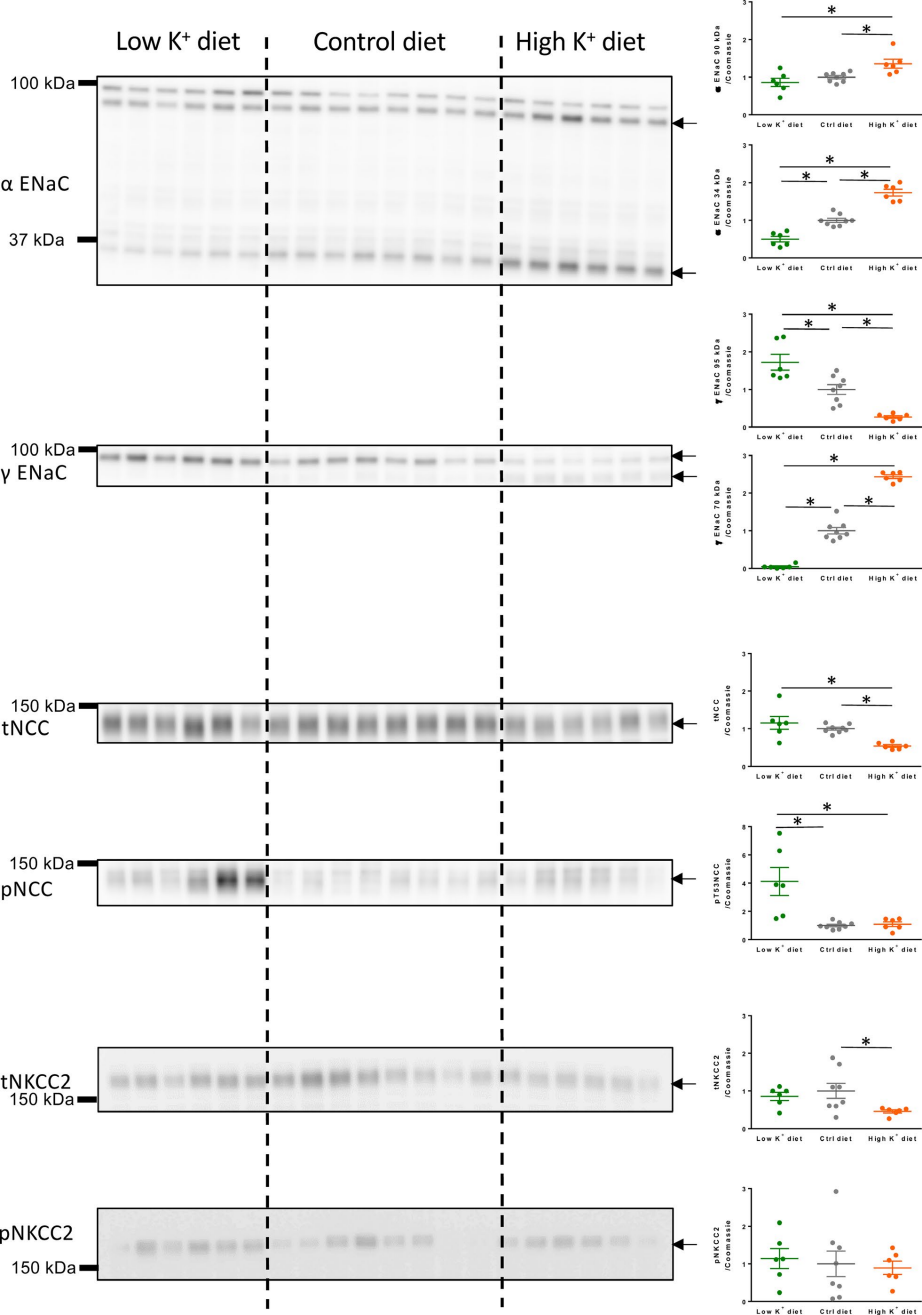
Semi‐quantitative Immunoblots of full length (~90 kDa) and cleavage activated (~34 kDa) α‐ENaC, full length (~95 kDa) and cleavage activated (~70 kDa) γ‐ENaC, total and phosphorylated NCC and total and phosphorylated NKCC2 in kidneys from mice kept on low, normal and high K^+^ diet. Reduced cleavage activation of both α‐ENaC and γ‐ENaC was detected in the low K^+^ diet group whereas high K^+^ diet augmented cleavage activation of α‐ENaC and γ‐ENaC. K^+^ restriction augmented the amount of phosphorylation‐activated NCC. Arrows mark quantified bands. Panels with equal variance between groups were analyzed by one‐way ANOVA. Panels with unequal variance between groups were analyzed by Brown‐Forstythe and Welch ANOVA. Both were followed by Bonferroni's multiple comparisons test. *n* = 6–8. *indicates *p* < .05

Semiquantitative western blotting of distal tubular Na^+^ absorptive proteins showed a clear regulation pattern as a function of the K^+^ content in the diets (Figure 4). Full‐length (~90 kDa) α‐ENaC was increased by dietary K^+^ loading. More importantly, the proteolytically cleaved (~34 kDa) and thus activated α‐ENaC (Ergonul et al., 2006) was significantly reduced in kidneys from K^+^ restricted mice and increased in kidneys from K^+^ loaded mice as compared to kidneys from control mice. The amount of full‐length (~95 kDa) γ‐ENaC was inversely related to the dietary K^+^ content, thus it was significantly reduced in kidneys from K^+^ loaded mice and augmented in kidneys from K^+^ restricted mice. Of functional importance, the proteolytically activated γ‐ENaC (Ergonul et al., 2006) (~70 kDa) was strongly reduced in kidneys from the K^+^ restricted mice and more than 2‐fold increased in kidneys from K^+^ loaded mice when compared to control kidneys.

Dietary K^+^ loading reduced the expression of total NCC and K^+^ restriction led to a significant increase in phosphorylation‐activated NCC at phosphor‐threonine residue 53.

The amount of total protein of the furosemide target NKCC2 was significantly reduced in kidneys from the K^+^ loaded mice. However, we detected no regulation of the phosphorylation‐activated NKCC2 at phosphor‐threonine residues 96 and 101.

In summary, these data from mice fed chow with varying K^+^ content support the notion that baseline ENaC expression is the hallmark in the determination of the magnitude of furosemide‐induced kaliuresis.

### 3.5 Benzamil‐induced natriuresis is reduced under low Na^+^ diet conditions

**FIGURE 5:**
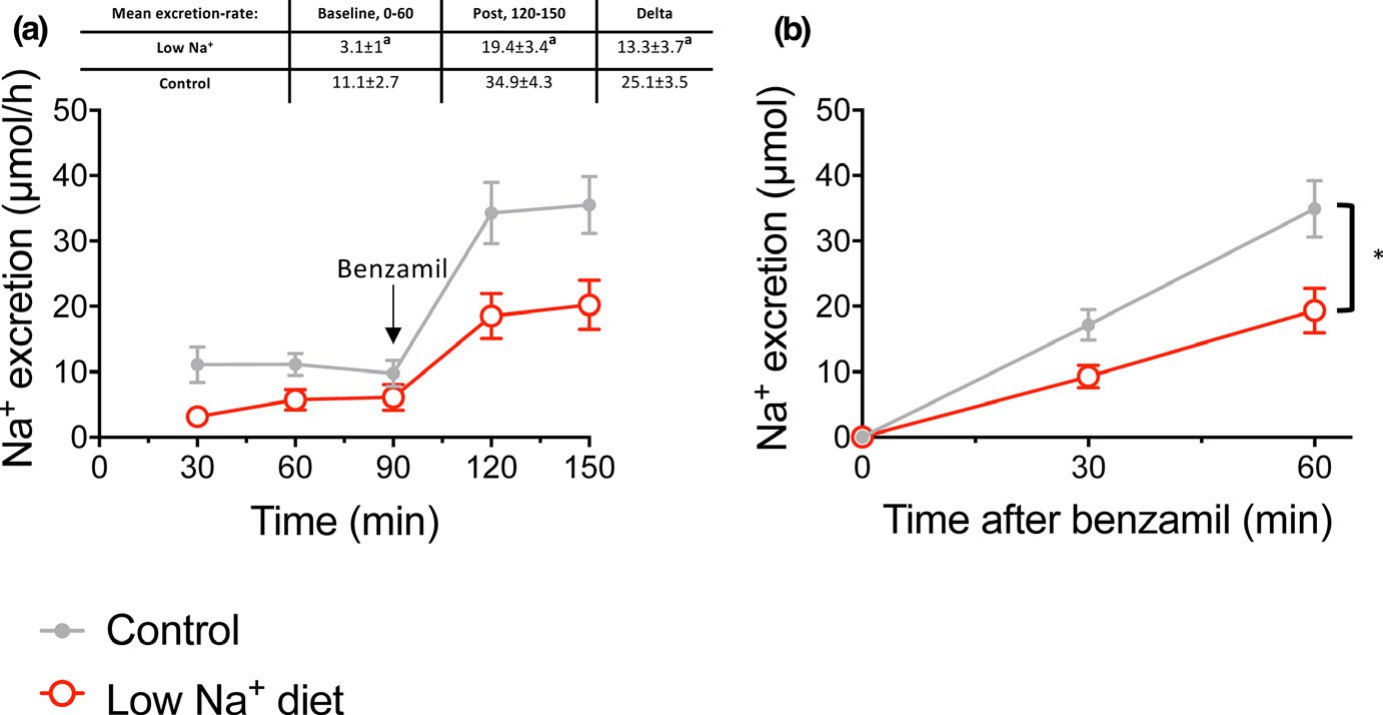
Benzamil‐induced natriuresis in mice kept on control‐ or a low Na^+^ diet. (a) Time‐dependent Na^+^ excretion 90 min prior (baseline) and 60 min post benzamil injection in control‐ and dietary Na^+^ restricted mice. Baseline natriuresis was significantly reduced in the Na^+^ restricted mice as compared to control. Benzamil induced a natriuretic response in both dietary groups. The insert depict the difference between the mean Na^+^ excretion prior to benzamil injection versus the mean K^+^ excretion 30 min post benzamil injection. The benzamil‐induced increased in natriuresis was significantly reduced in the Na^+^ restricted mice as compared to control mice. (b) Cumulative natriuresis post benzamil injection. Cumulated Na^+^ excretion post benzamil treatment was decreased in Na^+^ restricted mice. Differences between the two diet groups were evaluated by comparing the mean 60 min. baseline values, the difference between mean pre‐and post‐treatment values and the cumulative excretion after benzamil treatment and analyzed by *t*‐test. *n* = 9–10

From the above‐presented data, it is apparent that efficient urinary K^+^ elimination requires concurrent molecular ENaC activation and Na^+^ delivery to the ENaC‐expressing tubular segments. This advocates that in the native kidney ENaC could be molecularly activated (eg, augmented expression or proteolytic activation) but functionally silenced due to a lack of Na^+^ as substrate. Benzamil‐induced natriuresis is a measure of functional renal ENaC activity (Sorensen et al., 2019). We measured the natriuretic effect of a single dose of benzamil in mice kept on the control and the ENaC‐stimulating low Na^+^ diet. Baseline natriuresis measured during the first 30 min was lower in Na^+^ restricted mice compared to controls. Interestingly, the benzamil‐induced increase in natriuresis as well as the cumulated Na^+^ excretion post‐furosemide treatment were significantly greater in the control mice as compared to the Na^+^ restricted mice (Figure 5). This apparent reduction in functional ENaC activity is in contrast to the enhanced molecular ENaC activation in the Na^+^ restricted mice (Figure 2). Notably, these mice displayed increased NCC phosphorylation (Figure 2) suggesting high functional NCC activity and normal to high NKCC2 activity (Figure 1) that could restrict tubular Na^+^ delivery as the substrate for ENaC‐mediated Na^+^ reabsorption.

## 4 DISCUSSION

In the present study, we show that furosemide‐induced kaliuresis is increased in dietary regimes that stimulate ENaC activity and reduced in dietary regimes that impede ENaC activity (schematic illustration in graphical abstract). Thus, K^+^ loss is minimal when furosemide is given during conditions with low endogenous aldosterone levels. This is in alignment with a study by Wilcox et al. (1984) showing that furosemide‐induced K^+^ loss can be augmented directly by aldosterone.

Renal adaptations to altered electrolyte intake are multi‐segmental and involve adaptations of ion and fluid handling in the proximal tubular segments as well as in TAL and distal segments. Thus, functional data from the intact organism are very complex in nature. The final obtained data following furosemide administration will be influenced by homeostatic regulated tubular function.

With this caveat taken into account, we still see great value in these integrated physiological data.

Several studies (Ergonul et al., 2006; Frindt et al., 2008; Frindt & Palmer, 2009; Todkar et al., 2015), including data from our own lab (Jensen et al., 2016), have addressed Na^+^ and K^+^ intake‐induced regulation of ENaC cleavage and NCC phosphorylation. The data presented in the current study display some discrepancies to most previous studies. One key difference could be related to the conditions of tissue harvesting. In the current study, kidneys were harvested from mice post the functional measurements. It has the strength that we compare both functional and molecular data from the same individual animals. However, the mice have been without access to the experimental diets and anesthetized for 2 hr prior to tissue harvesting.

ASDN‐mediated K^+^ secretion is augmented by increased Na^+^ delivery to the ENaC‐expressing segments (McDonough & Youn, 2013). More Na^+^ as substrate for ENaC is assumed to increase ENaC‐mediated Na^+^ currents that depolarize the apical membrane. The depolarized membrane augments the driving force for K^+^ efflux and thus propels K^+^ excretion (Welling, 2013). Accordingly, the K^+^ wasting effect of loop diuretics is explained by their prominent natriuretic effects. This “Na^+^ delivery model” is also implemented in the concept of plasma K^+^ as a direct regulator of ASDN‐mediated Na^+^ reabsorption. Plasma K^+^ determines whether Na^+^ reabsorption should be primary electroneutral (via NCC) or electrogenic (ENaC‐mediated). Physiologically, the DCT and the CD cells directly monitor the interstitial K^+^ concentration (Penton et al., 2016; Terker et al., 2015). In the DCT, a reduction or an increase in the basolateral K^+^ concentration results in very rapid activation or inactivation of NCC, respectively. Consequently, the amount of Na^+^ delivered to the CD can be modulated by turning electroneutral NCC‐mediated Na^+^ reabsorption on or off. The principal cells of the CD respond to augmented interstitial K^+^ concentration with a rapid, direct, and aldosterone‐independent activation of ENaC‐mediated electrogenic Na^+^ reabsorption (Sorensen et al., 2019). Thus, the kidney responds with a complex intrinsic regulatory response to promote K^+^ elimination or conservation based on the plasma K^+^ concentration. Opposed to the renal autoregulatory response to elevated plasma K^+^ concentration, furosemide does not acutely activate ENaC. Thus, the magnitude of ENaC‐mediated Na^+^ reabsorption and ensuing ENaC‐dependent K^+^ secretion post/following the acute furosemide application are reliant on the endogenous a priori ENaC activity.

The concept that renal K^+^ excretion depends on both adequate molecular ENaC activity and Na^+^ delivery to the ASDN finds support in previous studies. Early tubule microperfusion studies in rats showed that a 2‐fold augmentation of distal tubular luminal Na^+^ concentration from 46 to 94 mM hardly influenced K^+^ excretion (Good & Wright, 1979). In a later study, the same experimenters confirmed that changing the distal tubular luminal Na^+^ concentration from 96 to 34 mM was also without any effect on the secretory K^+^ flux (Good et al., 1984). In contrast, a further reduction of luminal Na^+^ concentration from 34 to 10 mM led to a 50% reduction of the secretory K^+^ flux (Good et al., 1984). All animals used in these studies were kept on standard chow and therefore likely displayed relatively low endogenous aldosterone and ENaC activity levels. This supports the concept of a saturable ENaC‐dependent K^+^ secretion. In alignment with this concept, a more recent in vivo study addressed the electrolyte excretion after acute pharmacologic NCC inhibition with thiazides (Hunter et al., 2014). This maneuver is comparable to furosemide administration as it increases Na^+^ delivery to the ENaC‐expressing tubular segments without direct acute effects on ENaC. In control mice, with assumable low basal ENaC activity, no acute thiazide‐induced kaliuresis was detected. Interestingly, in mice kept a few days on a low Na^+^ intake, a thiazide‐induced kaliuresis could be detected (Ayasse et al., 2017; Hunter et al., 2014). In contrast, Li et al. (2017) reported a significant thiazide‐induced kaliuresis in mice kept on standard rodent chow. This discrepancy might relate to strain and housing‐specific variation in baseline RAAS/ENaC activity.

Distal tubular Na^+^ reabsorption undergoes switches dynamically between an electroneutral and an electrogenic modus depending on the need for predominantly Na^+^ conservation or K^+^ excretion (during variable degrees of Na^+^ conservation). This implies that during conditions where both Na^+^ and K^+^ conservations are desired, molecular‐activated ENaC might lack Na^+^ as substrate, since upstream NCC is highly active, and therefore leaves molecular‐activated ENaC channels functionally inactive. We tested this hypothesis experimentally by measuring benzamil‐induced natriuresis in Na^+^‐depleted mice. In states of volume depletion (eg, Na^+^ depletion), the renin‐angiotensin‐aldosterone cascade is activated. Angiotensin‐II is known to directly activate NCC (Wu et al., 2018), and is thus a stimulator of Na^+^ reabsorption upstream to the ENaC‐expressing segments. Concurrently, ENaC is also activated directly by aldosterone. Interestingly, when we measured benzamil‐induced natriuresis, as a functional measure of ENaC activity, it was significantly lower in the low Na^+^ diet group, as compared to the control diet group. Hence, despite clear molecular upregulation of ENaC activity, confirmed by western blotting, ENaC‐mediated Na^+^ absorption is not increased during states of Na^+^ conservation. The silenced ENaC molecules can, however, become functionally activated when Na^+^ delivery to the site of ENaC expression is increased, for example, by furosemide administration. This concept, that sodium delivery to ENaC‐expressing tubular segments is a determent of functional ENaC activity, is supported by studies of Udwan et al. (2017) and Frindt et al. (2018), highlighting the importance of differentiating between molecular and functional ENaC activity.

That we report that a K^+^‐rich diet enhances furosemide‐induced kaliuresis seems to contradict the findings from Wang et al. (2019), who reported that under an alkali K^+^‐rich diet furosemide becomes K^+^ sparing. A number of experimental differences may contribute to the contrary conclusion between the two studies. Besides the obvious difference in the dietary regime, where we load with KCl and Wang and co‐authors load with HCO_3_^−^ and citrate as anions, the time and route of furosemide load are also different between the two studies. We determine the very acute effect of (the first hour) IP‐injected furosemide, whereas Wang et al. (2019) address more chronic effects (1–7 days) of furosemide administrated via drinking water. Under all experimental conditions, we observe a strong diuretic effect of IP furosemide administration, which is in contrast to the minor diuretic response of furosemide that Wang et al. (2019) report.

Our high time resolution of the furosemide effects reveals that furosemide‐induced natriuresis and kaliuresis seem to display a slower onset in control animals compared to mice dietary loaded with either Na^+^ or K^+^ or mice replete for Na. We have discussed this issue both internally and with colleagues in the field of renal physiology. We have no satisfying conceptual explanation for this finding.

In this study, we addressed the acute kaliuretic effect of furosemide. Long‐term clinical use of furosemide in the treatment of congestive heart failure (Felker, 2012) is in most cases associated with urinary K^+^ loss and hypokalemia (Aldahl et al., 2017). From the results of this study, one would expect molecular ENaC upregulation to explain this. Indeed, chronic furosemide administration causes intravascular hypovolemia and acts as a stimulus for aldosterone secretion and thus ENaC upregulation (Garty & Palmer, 1997).

In summary, the magnitude of acute furosemide‐induced kaliuresis depends heavily on the baseline molecular activation of ENaC. This indicates that an estimation of furosemide's acute kaliuretic effect requires an assessment of the baseline ENaC activity level. In a clinical setting, these considerations might be important before administrating furosemide to patients with acute volume overload or hyperkalemia.

## CONFLICT OF INTEREST

The authors declare no conflict of interest.

## AUTHOR CONTRIBUTIONS

NA, JL, and MVS generated the general hypothesis and research concept. NA, PB, and MVS conducted the experiments and analyzed the data. NA and MVS drafted the manuscript. All authors critically revised and approved the final version of the manuscript.

## Supporting information



Fig S1

Fig S2
